# Popular Fallacies*Read before the Kansas State Homœopathic Medical Society, May 2d, 1895, C. F. Menninger, M. D., President.

**Published:** 1895-08

**Authors:** C. F. Menninger

**Affiliations:** Topeka, Kansas


### POPULAR FALLACIES.*

C. F. Menninger, M. D., Topeka, Kansas.

There is no department of science but what has its popular fallacies. A little knowledge, coupled with a vast amount of ignorance, has time and again been the cause of manufacturing and perpetuating many popular fallacies. There was a time when the science of geology was said to overthrow the teach-

---

* Read before the Kansas State Homœopathic Medical Society, May 2d, 1895, C. F. Menninger, M. D., President.

ings of Holy Writ. To believe in the teachings of advanced scholars in geology was to renounce all allegiance and faith in the Scriptures. But as the rising sun of intelligence gradually sent its illuminating rays into the minds of all people, this mist and fog has cleared away, and now all see and believe that the story of Genesis is written in the strata of the earth.

So ignorance concerning Homœopathy has caused the rise and prevalence of many, very many, fallacious doctrines and ideas. Nor are these in the minds of the common people alone, but also in those who, by reason of most ample opportunities, might know better. The shafts of ridicule are still being hurled against the impregnable truths of *similia*. They sting for the moment of contact; they make a clatter of noises as they strike, and then break and fall upon themselves, but they do not conquer. Truth prevails. How often do we, as practitioners of Homœopathy, have it told us how Johnnie one day, when his mother was out, got into the homœopathic medicine case and ate up nearly all the sugar medicine, and it did not hurt him at all?

But it is not the fallacies that are common in the rank and file of the people at large of which I wish to speak, but of some that are found within the sacred precincts of the temple itself. And here, too, ignorance, or, rather, a vast want of definite knowledge, is again the causative factor. To discuss "why this want of knowledge" would lead us into a very interesting by-path of discussion which we must leave for some future effort, for we would become too prolix and tiresome to attempt all. I wish merely to call your attention to a few of these, and to drop a remark or two upon their general character, effect, and causation.

The first that comes to mind, and one that is the cause of many injurious effects upon Homœopathy, is the quite general belief by physicians of the utter uselessness of every student of Homœopathy studying and having a good knowledge of the sciences of literature and of art—that is, to have a thorough college education prior to his entry upon the study of medicine. The arguments made, that he would not have time to do so, or that it would unfit him for medicine, or that it would be too expensive and could not be accomplished by but very few, or that it would not be of any benefit to him, or that we have hundreds of successful practitioners, good homœopaths, who have not done so—all this argument is specious and fallacious, and cannot stand up long enough to be controverted.

The founders and promoters of Homœopathy, those whose names are to be found upon and in the text and reference-books of all good homœopathic physicians of to-day, were men who had taken full university courses, many of them graduating at the head of their classes. They then took up the study of medicine and got all that was obtainable in the universities of the continent. Would that all followers of Homœopathy had been such students! Old-school medicine was weighed and found wanting. These men adopted Homœopathy, and it is to their culture, intelligence, and knowledge that we owe the existence of Homœopathy. When we are sorely tried and puzzled with a severe case, it is to their words of wisdom, advice, and counsel that we flee for help and guidance. Their works are our compass and our rudder in every troublous sea. The day must and will come when medicine will be a post-graduate study in this country, as it is in Europe.

Another fallacy that is too popular in the ranks of the homœopathic school, and one that to every true homœopath is the most alarming of all evils within our body politic, is the idea of the utter inutility or uselessness of studying *The Organon* and *Chronic Diseases*, both in college and in practice. There are thousands of physicians in our school who have never read *The Organon*, and many more who have never read the *Chronic Diseases*. Thousands, too, who did read it, or had it read to them in college, have never looked at or within it since. Perhaps I am a pessimist or misanthrope, and make it appear worse than it really is, but it is an alarming state, however you view it.

He who questions these assertions needs only to look over the libraries of homœopathic physicians. He who doubts needs only to talk to homœopathic practitioners on this subject. He who has misgivings as to the truth of the prevalence of this popular fallacy needs but to examine critically the courses of study of the homœopathic medical colleges of this country. Here this subject ought to be taught if they are going to educate *homœopaths*. In what colleges of the United States do we find *The Organon* and the *Chronic Diseases* as a regular subject for lecture once a week, not to say oftener? Echo answers, "where?" In what homœopathic colleges are the students made familiar with the masterpieces of Dunham, or of Hering, or of Rane? And need we bring forth any argument to establish the fact that these things need to be taught weekly and oftener? Where are our Herings, or Lippes, or Dunhams to-day? Their absence is only to be accounted for in the existence and too prevalent practice of this fallacious doctrine.

One other popular fallacy that is very generally held by homœopaths of to-day is that there is nothing in the higher potencies, as the 30th, 200th, 1M, 10M, and CM. I have not much to say on this except that homœopaths are among the strangest mortals into which the Creator ever put the breath of life. They cannot tell how the 3x will cure a patient, but they would swear to it that it has done so for them many a time. They cannot tell *how*, but they have tried it and found it to be true by actual demonstration. They cannot prove it in any other way to one who would laugh at and ridicule the idea. Yet they in turn laugh at and ridicule him who says the 1M or CM will do so. They say they have no faith in it. Did any one have faith in the 3x before they tried it honestly, earnestly, and perseveringly? No, never, or else every allopath would be a homœopath by this time. No scientific theorum or explanation has ever been found that will explain the action of the potentized medicine. If one is ever found that will satisfactorily explain the how and the why of the 3x potency it will also explain the 30th or the 1M. One thing is a settled fact that ought to knock to atoms *this* popular fallacy, and that is that the founders and promoters of Homœopathy—they who made it what it is to-day, they to whom all flee in times of trouble—all used the higher potencies.

